# Breast cancer risk in relation to urinary and serum biomarkers of phytoestrogen exposure in the European Prospective into Cancer-Norfolk cohort study

**DOI:** 10.1186/bcr1995

**Published:** 2008-04-17

**Authors:** Heather Ward, Gaelle Chapelais, Gunter GC Kuhnle, Robert Luben, Kay-Tee Khaw, Sheila Bingham

**Affiliations:** 1 MRC Centre for Nutrition and Cancer, Department of Public Health and Primary Care, Institute of Public Health, University of Cambridge, Strangeways Research Laboratory, Wort's Causeway, Cambridge, CB1 8RN, UK; 2 MRC Dunn Human Nutrition Unit, Wellcome Trust/MRC Building, Hills Road, Cambridge CB2 0XY, UK; 3 European Prospective Investigation of Cancer, Institute of Public Health, University of Cambridge, Strangeways Research Laboratory, Wort's Causeway, Cambridge CB1 8RN, UK

## Abstract

**Introduction:**

Phytoestrogens are a group of compounds found in plants that structurally resemble the hormone oestradiol, and thus have the potential to act as oestrogen agonists or antagonists. Their potential effects may alter the risk of breast cancer, but only a limited range of phytoestrogens has been examined in prospective cohort studies.

**Methods:**

Serum and urine samples from 237 incident breast cancer cases and 952 control individuals (aged 45 to 75 years) in the European Prospective into Cancer-Norfolk cohort were analysed for seven phytoestrogens (daidzein, enterodiol, enterolactone, genistein, glycitein, *o*-desmethylangolensin, and equol) using liquid chromatography/mass spectrometry. Data on participants' diet, demographics, anthropometrics, and medical history were collected upon recruitment. All models were adjusted for weight, fat and energy intake, family history of breast cancer, social class, analytical batch, and factors related to oestrogen exposure.

**Results:**

Urinary or serum phytoestrogens were not associated with protection from breast cancer in the European Prospective into Cancer-Norfolk cohort. Breast cancer risk was marginally increased with higher levels of total urinary isoflavones (odds ratio = 1.08 (95% confidence interval = 1.00 to 1.16), *P *= 0.055); among those with oestrogen receptor-positive tumours, the risk of breast cancer was increased with higher levels of urinary equol (odds ratio = 1.07 (95% confidence interval = 1.01 to 1.12), *P *= 0.013).

**Conclusion:**

There was limited evidence of an association between phytoestrogen biomarkers and breast cancer risk in the present study. There was no indication of decreased likelihood of breast cancer with higher levels of phytoestrogen biomarkers, but the observation that some phytoestrogen biomarkers may be associated with greater risk of breast cancer warrants further study with greater statistical power.

## Introduction

It has been proposed that phytoestrogens – dietary compounds that structurally resemble the hormone oestrogen – are associated with the development of breast cancer due to their potential to act as oestrogen agonists or antagonists [1-3]. Phytoestrogens are a complex group of compounds widespread at low levels in most western plant foods. There are two main classes: the isoflavones (daidzein, genistein, and glycitein), present at high levels in soya products [4]; and the lignans (secoisolariresinol, matairesinol, pinoresinol, and lariciresinol) found in wholegrain cereals, fruits and vegetables, and flaxseed [5,6]. *O*-Desmethylangolensin (O-DMA) and equol are metabolites of daidzein, and enterodiol and enterolactone are metabolites of lignans; the parent compounds are metabolised by the gut flora. Both precursors and metabolites can be measured in biological fluids [7].

The proposed protective mechanisms of phytoestrogens include stimulation of apoptosis, antioxidant activity, inhibition of enzymes associated with carcinogenesis and steroid metabolism, and competitive binding to oestrogen receptors (ERs) [8-12]. Phytoestrogens, however, may be more likely to demonstrate antiestrogenic behaviour when endogenous oestrogen is high, and to exhibit oestrogenic activity when circulating endogenous oestrogen is low [2,13,14]; phytoestrogens may therefore be more strongly associated with oestrogen-sensitive breast cancers. Of the phytoestrogens, the metabolite equol is more oestrogenic in *in vitro *assays than its precursor daidzein, although only about one-third of individuals are reported to be capable of producing equol [7,15].

Serum and urinary biomarkers of phytoestrogen exposure serve as an alternative indicator of dietary intake, and are advantageous in that they are not reliant on self-reported dietary information or on food composition databases for phytoestrogen quantification. Furthermore, the use of biomarkers reflects the variation across individuals in terms of gut microflora, which is responsible for the production of equol and O-DMA from its parent compound daidzein, and is influenced by stress, diet, bowel disease, genetics, and antibiotic use among other factors [15]. Studies to date have generally included a limited range of phytoestrogen biomarkers, with the majority of studies focused solely on enterolactone [16-21]. Depending on the phytoestrogen under examination, risk for breast cancer has appeared to be increased by [17,20-22], to be decreased by [16,17,19,20,23-26] or not to be associated with [18,20-25,27] higher serum or urinary levels. Previous assays in biological fluids have often suffered from complex, multistage sample preparation, poor precision and substantial limitations in the number of these complex analytes that could be assessed at low levels found in biological fluids [15,22].

Using newly developed highly sensitive and rapid methods for the analysis of phytoestrogens by mass spectrometry incorporating triply ^13^C-labelled standards for quantitation, we have previously shown that phytoestrogens in plasma and serum are closely correlated [22] and that odds ratios for equol exposure were significantly increased in the relatively small European Prospective into Cancer (EPIC)-Norfolk cohort study (97 cases with serum and urine data, and 17 cases with urine data only) [22]. In the Dutch arm of the EPIC study, however, an inverse effect for plasma genistein (odds ratio = 0.68, 95% confidence interval = 0.47 to 0.98) was detected among 383 cases and 383 control individuals, despite the use of the same study design and laboratory methods as the EPIC-Norfolk cohort [25]. We have therefore enlarged the EPIC-Norfolk study to include a total of 237 cases matched with double the numbers of control individuals. To our knowledge, this is the largest study to date utilising accurate and sensitive mass spectrometry methodology to assess phytoestrogen exposure.

## Materials and methods

Between 1993 and 1997, men and women aged 45 to 75 years were recruited from general practitioners' databases for the EPIC-Norfolk study [28]. A health and lifestyle questionnaire was administered by mail, collecting data on smoking, alcohol consumption, social class, basic family medical history, exercise, and reproductive history [28]. Participants completed 7-day diet diaries that were entered into an inhouse dietary assessment software program – Data into Nutrients for Epidemiological Research – to calculate average daily nutrient intakes [29]. Height and weight measurements and blood samples were collected as part of a health check conducted by nurses. Spot urine and plasma serum were stored at -20°C and -80°C, respectively, prior to analysis. Questionnaires and health checks were completed by 25,639 healthy adults, 14,032 of whom were women. The study was approved by the Norwich District Health Authority Ethics Committee, and all participants gave signed informed consent.

The International Classification of Diseases, Injuries, and Causes of Death was applied for the classification of incident cases of invasive breast cancer (ICD9 174, ICD10 C50C). Between 1993 and July 2006, there were 237 incident cases of invasive breast cancer identified through the East Anglia Cancer Registry, diagnosed a minimum of 12 months after recruitment in the EPIC-Norfolk cohort.

Cases were matched with four control individuals based on age (± 3 years) using the entire EPIC-Norfolk cohort prior to the analysis of serum and urine phytoestrogen data. Serum and urine samples were not available, however, for all of the cases and control individuals; as a result, the number of controls varied from one to four per case. For the present analysis of phytoestrogens, therefore, the data were treated as frequency matched and adjusted for age.

All 237 cases and 952 control individuals had completed a food diary and had at least one blood or urine sample available for analysis. The ER status of tumour samples was also obtained from the East Anglia Cancer Registry. ER-positive tumours were detected among 115 cases, ER-negative cases were detected among 27 cases, and ER status information was not available for 95 cases. Seventy-four per cent of cases without ER status were from the first batch; this was probably due to the fact that ER testing was not routinely conducted until 1999. Plasma oestradiol was available for 153 cases and 603 control individuals using methods for estimating plasma oestradiol published previously [30].

Serum samples, drawn at the health check, were analysed for daidzein, genistein, glycitein, O-DMA, equol, enterodiol and enterolactone. Samples were analysed as described previously [22]. Briefly, phytoestrogen metabolites were enzymatically deconjugated and extracted using Strata C18-E SPE cartridges (Phenomex, Macclesfield, UK). Following extraction, the metabolites were dried under vacuum, redissolved in 40% aqueous methanol and analysed using liquid chromatography/mass spectrometry (Waters Quattro Ultima; Waters, Manchester, UK). Samples were quantified using triply ^13^C-labelled internal standards. The average intra-assay coefficient of variation for serum analytes ranged from 2.8% (enterolactone) to 20.0% (glycitein). Limits of detection for serum phytoestrogens ranged from 0.04 ng/ml for daidzein to 0.11 ng/ml for equol, corrected for the concentration of sample [22]. The 284 serum samples that had been quantified for the previous study [22] were designated Batch 1 for statistical analysis, leaving 905 serum samples designated as Batch 2.

Spot urine samples were analysed for the same phytoestrogens as the serum samples. Three hundred and thirty-three urine samples were analysed using isotope dilution gas chromatography/mass spectrometry, wherein dried extracts were derivatised to their trimethylsilyl derivatives and were designated Batch 1 as before. The remaining 731 samples (Batch 2) were analysed by liquid chromatography/mass spectrometry, in which the dried extracts were redissolved in 40% aqueous methanol and were analysed as above. The limits of detection with gas chromatography/mass spectrometry after correction for dilution of sample ranged from 1.8 ng/ml (enterodiol) to 8.0 ng/ml (enterolactone). For liquid chromatography/mass spectrometry, the limit for detection ranged from 0.08 ng/ml for daidzein to 0.22 ng/ml for equol. The intra-assay coefficient of variation for urinary phytoestrogens ranged from 2.3% (daidzein) to 6.8% (equol). Details of the quality assurance and methodology have been published elsewhere [31].

### Statistical methods

For case–control comparisons of potential confounders, a chi-square test was applied to categorical variables and an unpaired Student *t *test was conducted for continuous variables (log-transformed). To be retained in the log transformation, the value 0.011 was assigned arbitrarily for phytoestrogen levels below the level of reliable quantification, and the value 0.01 was when the compound was not detected at all. Pearson correlation coefficients were calculated to ascertain the relationship between log-transformed urinary and serum measures of each phytoestrogen. The Wilcoxon–Mann–Whitney test was applied to determine whether the probability distribution for serum and urinary phytoestrogens differed between cases and control individuals.

Odds ratios for breast cancer risk were calculated using unconditional logistic regression. Summary variables of total lignans (enterodiol and enterolactone) and total isoflavones (daidzein, genistein, O-DMA, equol, and glycitein) were created and analysed in conjunction with individual phytoestrogens. Phytoestrogen values were log_2_-transformed, so the estimates of risk correspond to a doubling in phytoestrogen level [32]. All models were adjusted for batch and for variables related to oestrogen exposure: age at menarche (continuous), parity (ordinal), breastfeeding (categorical), history of oral contraceptive use (categorical), menopausal hormone therapy use (categorical), and menopausal status (categorical). Additional potentially confounding variables were retained in the analysis if the likelihood ratio test yielded a significant *P *value or if the β values for the phytoestrogen in the model were modified by 10% or more when added individually to the model. This approach identified weight (continuous), fat (continuous), energy (continuous), family history of breast cancer (categorical), and social class (categorical) as covariates. Smoking status (categorical), education (categorical), fibre intake (continuous), height (continuous), and physical activity level (categorical) were found to be nonsignificant covariates and were therefore not included in the final models. Among subjects with oestradiol data available, adjustment for oestradiol (continuous) did not alter the β values for the phytoestrogens under examination and was therefore not included in the final models.

Covariates were missing for fewer than 5% of the sample unless specified otherwise; subjects with missing covariate data were deleted from the analysis. Analyses were repeated for cases with tumours that were identified as ER-positive and their controls. The small number of participants with ER-negative tumours precluded analysis of this subgroup. To further explore the role of oestrogen, the sample was stratified by menopausal status (group 1, premenopausal and perimenopausal women; group 2, 2 years or more since last menstrual cycle), and analyses were re-run on the subgroup with oestradiol data available.

All analyses were conducted with SAS software (version 8.02; SAS Institute, Inc, Cary, NC, USA). All *P *values are two-sided; values less than 0.05 were considered significant, with marginal significance noted below 0.1.

## Results

Demographic and lifestyle characteristics of the 237 breast cancer cases and 952 control individuals are presented in Table 1. The mean length of follow-up was 9.5 years (standard deviation, approximately 3.1 years), representing 11,261 person-years. In the population of analysis, 80% were postmenopausal and 65% reported that they had breastfed (Table 1). The frequency of family history of breast cancer, social class, and smoking did not differ across cases and controls. Relative to the control individuals, breast cancer cases reported higher mean weight, fat and energy intake, and marginally higher oestradiol levels. Use of oral contraceptive was distributed differently between cases and controls.

**Table 1:** Characteristics of the breast cancer case – control sample in the European Prospective into Cancer-Norfolk cohort

	Cases (n = 237)	Controls (n = 952)	*P *value^a^
Age (years)	58.5 (8.6)	58.7 (8.9)	0.791
Weight (kg)	69.2 (11.4)	67.7 (11.2)	0.068
Energy intake (kcal/day)	1745.9 (386.6)	1678.6 (385.2)	0.022
Fat intake (g/day)	66.5 (19.7)	63.5 (20.1)	0.038
Alcohol intake (g/day)	8.6 (12.1)	7.8 (11.2)	0.179
Oestradiol (pmol/l)^b^	173.1 (341.3)	136.9 (259.4)	0.066
Age at menarche (years)	12.8 (1.6)	13.0 (1.5)	0.058
Family history of breast cancer: yes	23 (10%)	74 (8%)	0.216
Breastfed children: yes	155 (65%)	617 (65%)	0.865
Number of children			
0	38 (16%)	117 (12%)	0.174
1	26 (11%)	134 (14%)	
2	103 (43%)	398 (42%)	
3	57 (24%)	218 (23%)	
4+	13 (6%)	85 (9%)	
Menopausal status^c^			
Premenopausal	28 (12%)	123 (13%)	0.569
Perimenopausal	15 (6%)	51 (5%)	
Postmenopausal 1	57 (24%)	195 (21%)	
Postmenopausal 2	137 (58%)	583 (61%)	
Menopausal hormone therapy use			
Current	63 (27%)	207 (22%)	0.256
Former	30 (13%)	118 (12%)	
Never	144 (61%)	627 (66%)	
Oral contraceptive use			
Current	9 (4%)	13 (1%)	0.041
Former	98 (41%)	419 (44%)	
Never	130 (55%)	520 (55%)	
Social class			
Professional/manager	105 (45%)	405 (42%)	0.620
Skilled nonmanual	46 (20%)	171 (18%)	
Skilled manual	51 (22%)	197 (21%)	
Semi-skilled/nonskilled	20 (9%)	112 (12%)	
Missing/unclassified	15 (4%)	67 (7%)	
Smoking status			
Current	26 (11%)	94 (10%)	0.822
Former	70 (30%)	297 (32%)	
Never	137 (59%)	550 (59%)	

Data presented as mean ± standard deviation or n (column percentage). ^a^*P *values obtained with a *t *test for continuous values and a chi-square test for categorical variables. ^b^Oestradiol data availability: serum phytoestrogens, 153 cases and 603 controls (51 cases and 185 controls among the oestrogen receptor-positive subgroup); urinary phytoestrogens, 141 cases and 543 controls (49 cases and 171 controls among the oestrogen receptor subgroup). ^c^Perimenopausal, <1 year since last menstrual cycle; postmenopausal 1, 2 to 5 years since last menstrual cycle; postmenopausal 2, 5 years or more since last menstrual cycle.

The median serum and urinary phytoestrogen levels are presented in Table 2. Cases and controls had significantly different distributions of serum equol, serum and urinary glycitein, and serum enterodiol; marginal differences were observed for serum enterolactone and urinary genistein. When the analysis was restricted to the ER subgroup, urinary enterolactone was distributed differently between cases and controls; marginal differences were detected for urinary glycitein and equol, and there were no differences observed for serum phytoestrogens. Of the precursor isoflavones, genistein was found in greater amounts than daidzein in serum, whereas daidzein was present in higher concentration in urine (Table 2); this different ratio has been noted previously [30]. After excluding those cases and controls with values below the level of quantification, the correlations between the serum values for each phytoestrogen (ng/ml) and the corresponding urinary values (μg/mmol creatinine) were as follows: enterodiol, 0.67; enterolactone, 0.79; equol, 0.63; daidzein, 0.75; genistein, 0.66; glycitein, 0.18; O-DMA, 0.72 – all correlations were significant at *P *= 0.003. Correlations including values below the level of quantification ranged from 0.27 to 0.58 (*P *< 0.0001).

**Table 2:** Mean and median serum and urinary phytoestrogen levels in the European Prospective into Cancer-Norfolk breast cancer case – control study

	Median	% detected	Median	% detected	*P *value^a^
Full study	Controls (n = 952)		Cases (n = 237)		
Serum (ng/ml)					
Genistein	5.00	91	4.77	93	0.608
Daidzein	2.00	86	1.98	91	0.206
Equol	0.01	29	0.01	33	0.005
*o*-Desmethylangolensin	0.10	52	0.10	59	0.284
Glycitein	0.01	29	0.01	40	<0.0001
Enterodiol	0.10	58	0.20	68	0.023
Enterolactone	5.83	98	5.00	98	0.088
Urine (μg/mmol creatinine)	Controls (n = 851)		Cases (n = 213)		
Genistein	5.71	84	6.47	86	0.071
Daidzein	14.82	92	14.63	94	0.134
Equol	0.011	47	0.011	52	0.403
*o*-Desmethylangolensin	0.92	73	0.58	75	0.486
Glycitein	0.62	69	1.29	79	<0.0001
Enterodiol	6.45	86	7.25	91	0.188
Enterolactone	112.70	97	102.07	97	0.366
Oestrogen receptor-positive subgroup	Controls (n = 381)		Cases (n = 112)		
Serum (ng/ml)					
Genistein	4.80	89	4.55	89	0.446
Daidzein	1.83	84	1.89	87	0.846
Equol	0.01	37	0.01	41	0.637
*o*-Desmethylangolensin	0.10	51	0.10	56	0.569
Glycitein	0.01	25	0.01	29	0.372
Enterodiol	0.10	59	0.17	61	0.645
Enterolactone	6.60	98	6.25	98	0.739
Urine (μg/mmol creatinine)	Controls (n = 344)		Cases (n = 102)		
Genistein	5.67	84	4.74	80	0.675
Daidzein	15.30	93	13.09	92	0.948
Equol	0.04	51	0.46	61	0.073
*o*-Desmethylangolensin	1.29	70	1.02	72	0.899
Glycitein	0.51	65	0.56	70	0.195
Enterodiol	6.62	85	8.82	91	0.075
Enterolactone	123.99	97	136.55	99	0.044

^a^*P *value from the Wilcoxon – Mann – Whitney test.

None of the serum phytoestrogens were associated with the risk of breast cancer, regardless of ER-positive tumour status. In the full study, an increased risk of breast cancer was observed with higher levels of total urinary isoflavones (Table 3). Among women with ER-positive tumours, the risk of breast cancer was associated with higher levels of urinary equol levels, and was marginally associated with greater exposure to total urinary isoflavones (Table 3). The observed associations were not affected by adjustment for oestradiol (data not shown). After stratification, a stronger association with breast cancer risk was detected for total urinary isoflavones among premenopausal and perimenopausal women (odds ratio = 1.30 (95% confidence interval = 1.04 to 1.64), *P *= 0.022), but was not significant among postmenopausal women (odds ratio = 1.01 (95% confidence interval = 0.96 to 1.13), *P *= 0.372); no change in results was observed for serum phytoestrogens (data not shown). The small number of premenopausal and perimenopausal women in the ER subgroup precluded stratification by menopausal status.

**Table 3:** Association between phytoestrogen biomarkers and breast cancer risk in the European Prospective into Cancer-Norfolk study

	Serum (ng/ml)			Urine (μg/mmol creatinine)		
	
	Odds ratio	95% confidence interval	*P *value	Odds ratio	95% confidence interval	*P *value
Full study	(219 cases/891 controls)			(198 cases/797 controls)		
Total isoflavones	1.03	0.95 to 1.11	0.479	1.08	1.00 to 1.16	0.055
Total lignans	0.99	0.90 to 1.08	0.728	1.01	0.94 to 1.09	0.758
Daidzein	1.04	0.98 to 1.10	0.225	1.05	0.99 to 1.10	0.096
Equol	1.04	0.98 to 1.10	0.167	1.03	0.99 to 1.06	0.131
*o*-Desmethylangolensin	1.03	0.97 to 1.09	0.390	1.02	0.98 to 1.06	0.250
Genistein	1.00	0.94 to 1.05	0.911	1.01	0.97 to 1.05	0.706
Glycitein	1.03	0.97 to 1.10	0.345	1.03	0.98 to 1.07	0.248
Enterodiol	1.02	0.96 to 1.09	0.461	1.01	0.96 to 1.05	0.772
Enterolactone	0.98	0.91 to 1.05	0.564	0.99	0.94 to 1.04	0.718
Oestrogen receptor-positive subgroup	(105 cases/365 controls)			(95 cases/329 controls)		
Total isoflavones	1.01	0.91 to 1.12	0.818	1.09	0.97 to 1.22	0.154
Total lignans	1.02	0.89 to 1.17	0.774	1.12	0.99 to 1.28	0.083
Daidzein	1.05	0.97 to 1.13	0.260	1.03	0.96 to 1.10	0.468
Equol	1.01	0.93 to 1.09	0.887	1.07	1.01 to 1.12	0.013
*o*-Desmethylangolensin	1.05	0.96 to 1.14	0.314	1.04	0.98 to 1.10	0.192
Genistein	0.99	0.92 to 1.06	0.775	1.00	0.94 to 1.05	0.882
Glycitein	1.03	0.93 to 1.13	0.614	1.04	0.98 to 1.10	0.186
Enterodiol	1.00	0.92 to 1.09	0.990	1.04	0.98 to 1.11	0.235
Enterolactone	1.01	0.91 to 1.14	0.806	1.08	0.98 to 1.19	0.115

Values are log_2_-transformed and adjusted for weight, oral contraceptive use, menopausal hormone treatment, menopausal status, parity, menarche, breastfeeding, family history of breast cancer, daily intake of fat and energy, and batch.

## Discussion

Despite the biological plausibility of a proposed reduction in breast cancer risk with greater exposure to phytoestrogens [8-12], the present analyses provided no evidence that phytoestrogen biomarkers were associated with decreased breast cancer risk in the prospective EPIC-Norfolk cohort. The present study was well positioned to examine this association as the collection of biomarker samples prior to cancer diagnosis reduced the potential bias of health behaviour change in response to disease diagnosis. The mass spectrometry quantification method for samples used in the EPIC-Norfolk study offers greater specificity and sensitivity than that provided by time-resolved fluoroimmunoassay, the method commonly applied in prior phytoestrogen biomarker research [31], and was applied to a large variety of metabolites. Furthermore, with data available on other aspects of diet, anthropometric measurements, reproductive history, exogenous hormone use, and circulating levels of oestradiol, the present analyses were able to adjust for the majority of known risk factors for breast cancer risk. Nonetheless, the majority of the associations in the present analysis were null.

The predominant absence of associations between phytoestrogen biomarkers and breast cancer may be attributed to limitations specific to aspects of the study design. The half-life of daidzein, genistein, and equol has been reported as <12 hours [33]; the single samples of serum and urine drawn for analysis would therefore have been influenced by variation in the diet and in the gut microflora at the time of the health check and may not have been representative of usual intake. In a study with three yearly serum samples drawn over 2 years, the reliability coefficients ranged from 0.11 (daidzein) to 0.55 (enterolactone) [34]; these results suggest that a single sample is unlikely to represent usual intake. Furthermore, the exploration of the ER status in relation to phytoestrogen biomarkers and breast cancer risk may have been hampered by limited statistical power due to the small number of cases with these data available; the absence of ER status information for 40% of breast cancer cases in the present study raises the question of whether the associations observed for ER-positive cases would have appeared differently had that information been available for all subjects. Finally, it is possible that the biomarker samples were drawn outside the time period relevant for cancer development, as phytoestrogen intake prior to the maturation of the mammary gland may exert the greatest influence over breast cancer risk [35].

Increased risk with greater levels of urinary equol in the ER-positive subgroup of the present study was among the few significant associations detected; however, this association should be interpreted with caution as it was not also observed in the analysis of serum equol. This result is consistent with the previous study of women in the EPIC-Norfolk cohort, wherein breast cancer risk was positively associated with serum and urinary equol [22], and with the fact that equol has been identified as a highly oestrogenic compound [15]. Furthermore, it has been suggested that some phytoestrogens may be agonists rather than antiestrogens in the presence of relatively lower circulating levels of mammalian oestrogen [1,2], which may have been the state of the postmenopausal majority in the present study. Other studies have reported that serum equol was not associated with breast cancer risk [25], whereas urinary equol was associated with decreased risk [24]; however, neither study examined ER status as an effect modifier.

The multiple comparisons conducted in the present analysis may have created a false-positive finding for urinary equol in the ER-positive group. This methodological concern also applies to the marginally significant associations detected for total urinary lignans in the ER-positive cohort and for total urinary isoflavones in the full study. Additionally, the disparity between urinary and serum results may have been affected by fact that the proportion of participants with undetected values was generally higher for phytoestrogen biomarkers in serum compared with urine. Nonetheless, the evidence suggests that there may be circumstances where phytoestrogen exposure increases breast cancer risk, and thus deserves further study

The literature regarding a potential interaction between phytoestrogens and ER status in relation to breast cancer is limited and has not included a measure of equol. In a prospective Danish case–control study, plasma enterolactone was not associated with ER-positive breast cancer, but was associated with a decreased risk of ER-negative breast cancer; the authors noted that, with only 80 ER-negative cases, caution is warranted in interpretation of this result [36]. A prospective study of postmenopausal women in the Netherlands found no association between breast cancer and urinary genistein or enterolactone regardless of ER status [27]; again, equol was not assessed.

Studies of dietary measures of phytoestrogen in relation to ER-positive cancer risk have been limited by the quality of data available and by the range of phytoestrogens investigated. In a large French prospective study including 1,469 breast cancer cases, ER-positive and progesterone-positive breast cancer risk was inversely related to an estimate for intake of total dietary lignans (secoisolariresinol, matairesinol, pinoresinol, and lariciresinol) [37]. Two studies from the United States reported no association between dietary secoisolariciresinol and matairesinol and breast cancer, regardless of menopausal status or ER tumour status [38,39], with the exception of reduced risk of ER-negative breast cancer among premenopausal women with the highest level of intake [39]. Overall, further exploration is evidently required before definitive conclusions can be ascertained regarding the relationships between ER status, breast cancer risk, and phytoestrogens, particularly equol.

Only two prospective studies have examined a wide range of metabolites with the same methods as the present study [22,25]; all other studies were either retrospective or case–control in design [16,19,20,23,26] and/or utilised methods limited to one or two metabolites [16-21,27]. Among women in the case–control study from the EPIC-Dutch cohort, higher serum levels of genistein were related to a reduction in breast cancer risk, but there were no associations observed for glycitein, O-DMA, equol, enterodiol or enterolactone [25]. With the exception of daidzein, the geometric mean serum phytoestrogen levels were slightly higher in the EPIC-Norfolk cohort. The EPIC-Dutch and EPIC-Norfolk studies were comparable in terms of study design and the quantification of their serum samples, but dietary factors were evaluated and included as potential confounders only in the EPIC-Norfolk cohort. Overall, the studies were similar as the majority of findings were null.

Studies of phytoestrogen intake in western populations have yielded inconsistent conclusions on the nature of the relationship with breast cancer, with evidence of either decreased risk [37,40] or no association [41,42]. Accordingly, reviews of the epidemiological and experimental studies have not indicated a clear consensus regarding the nature of phytoestrogen exposure and breast cancer risk [43-47]. There is a great deal of heterogeneity among these dietary studies in terms of study design, the population examined, the type of phytoestrogens under study, the coverage of phytoestrogen-rich foods in the dietary questionnaires, and use of compiled data from various sources for the calculation of phytoestrogen levels in food [37,40]. Furthermore, phytoestrogens are a complex mixture of different compounds found at very low levels in foods and biological materials, and the analysis of foods is difficult due to matrix effects and the presence of substantial variation according to variety, crop, season and processing methods, and the requirement of sensitive methods able to detect low levels. In addition, phytoestrogens – particularly the lignans – are found in foods rich in other plant-protective compounds and it may not be possible to adequately correct for confounding by other food constituents, such as the dietary fibre, flavonoids, and antioxidants found in these foods.

Overall, the absence of strong associations between phytoestrogen biomarkers and breast cancer in the present study could indicate that the level of exposure was too low to yield an effect on cancer development. It has been noted previously that the intake of isoflavones is relatively low in the EPIC-Norfolk cohort [4]. The lower incidence of breast cancer that has been observed in Asian populations may be attributed to a higher intake of phytoestrogen-rich food, to higher levels of phytoestrogen biomarkers, and to production of equol relative to European populations [15,48-50]. Nonetheless, since it has been estimated by direct analysis of a representative sample of foods that there has been a small increase in intake of dietary isoflavones in the United Kingdom since the late 1980s [51], the relationship between phytoestrogens and breast cancer may differ across generations and ought to be monitored.

## Conclusion

The majority of the urinary or serum biomarkers for phytoestrogen levels were not associated with breast cancer risk in the EPIC-Norfolk cohort. The limited evidence of an association between urinary equol and ER-positive breast cancer cases warrants further study in subsequent examinations of breast cancer risk and phytoestrogen biomarkers. Overall, the present study does not provide sufficient insight into the nature of the relationship between phytoestrogens and breast cancer risk to warrant modification of phytoestrogen intake among those at risk for breast cancer.

## Abbreviations

EPIC = European Prospective into Cancer; ER = oestrogen receptor; O-DMA = *o*-desmethylangolensin.

## Competing interests

The authors declare that they have no competing interests.

## Authors' contributions

HW conducted the statistical analyses and wrote the manuscript. GC and GGCK conducted the laboratory analyses of serum and urine phytoestrogens, and contributed to the manuscript. RL participated in the statistical analysis and was responsible for follow-up and identification of cases. K-TK is a principal investigator of the EPIC-Norfolk study and contributed to the manuscript. SB led the study, was a principal investigator in the EPIC-Norfolk study, and drafted the manuscript with HW. All authors read and approved the final manuscript.
